# From Symptoms to Solution: A Diagnostic Challenge of Whipple’s Disease

**DOI:** 10.7759/cureus.81767

**Published:** 2025-04-05

**Authors:** Victoria Rutherford, Aneesa Afroze

**Affiliations:** 1 Internal Medicine, MercyOne Des Moines Medical Center, Des Moines, USA; 2 Infectious Disease, MercyOne Des Moines Medical Center, Des Moines, USA

**Keywords:** arthralgias, culture negative infective endocarditis, lymphadenopathy, malabsorption syndrome, tropheryma whipplei, whipple's disease

## Abstract

Whipple’s disease is caused by the bacterium *Tropheryma whipplei*. The classic presentation of this disease consists of arthralgias, weight loss, diarrhea, and abdominal pain. However, Whipple's disease can also have other manifestations across multiple organ systems, ranging from lymphadenopathy to pleuropulmonary disease to endocarditis to skin hyperpigmentation and even CNS involvement. We present the case of a 39-year-old male with multiple organ system manifestations of Whipple’s disease. He presented with a 50-pound unintentional weight loss, early satiety, arthralgias, chronic cough, pleural effusions, and lymphadenopathy on a CT scan. A transesophageal echocardiogram revealed vegetation on multiple valves, consistent with the diagnosis of endocarditis. Blood cultures showed no growth. Several labs were obtained for a complete work-up for culture-negative endocarditis. The serum polymerase chain reaction (PCR) was reported positive for *T. whipplei*. Gastric antral biopsy and inguinal lymph node biopsy showed periodic acid-Schiff (PAS)-positive macrophages, confirming the diagnosis of Whipple’s disease. The patient was treated with ceftriaxone for a four-week course, followed by Bactrim DS for one year. As was the case with this patient, Whipple’s disease can present a wide array of symptoms across multiple organ systems. It is often difficult to diagnose due to its rarity and broad range of non-specific symptoms. Physicians should maintain a high level of suspicion for this disease and include it in their differential diagnosis. A prompt diagnosis and appropriate treatment will yield better outcomes.

## Introduction

Whipple's disease is caused by infection with the gram-positive bacillus *Tropheryma* *whipplei*. This bacterium is commonly found in soil and water and can be transmitted via the fecal-oral route [1]. Asymptomatic carriage of the bacterium without disease development is common and can serve as a reservoir for human-to-human transmission [2,3]. The genetic factors of the host appear to be the main factors that contribute to the disease's development [1,2]. Whipple's disease is a relatively rare diagnosis, with about one to six new cases per 10,000,000 people per year worldwide [2]. Early detection of the disease is critical given the progressive and often fatal nature of untreated infection [2]. Infection with *T. whipplei *classically presents with the four cardinal symptoms of arthralgias, weight loss, diarrhea, and abdominal pain. However, Whipple's disease is also known to cause lymphadenopathy, CNS involvement, endocarditis, skin hyperpigmentation, pleuropulmonary disease, and malabsorption syndrome. In a patient with clinical suspicion for Whipple’s disease, more common causes of the presenting symptoms (inflammatory bowel disease (IBD), other infectious causes of diarrhea, connective tissue disease, HIV, tuberculosis (TB), hyperthyroidism) must first be ruled out. The next step in diagnosis is a quantitative polymerase chain reaction (PCR) on a non-invasive sample (feces, saliva, or blood), with a positive PCR test indicating a high likelihood of Whipple’s disease [2]. The final step in diagnosis consists of endoscopy with biopsy of the duodenum (or less commonly the gastric antrum, jejunum, and/or ileum), in which periodic acid-Schiff (PAS) positive staining of the biopsy sample is confirmatory for Whipple’s disease [2]. Our patient presented with two of the four cardinal symptoms (arthralgias and weight loss). He also had many other manifestations of Whipple's disease, including lymphadenopathy, endocarditis, chronic cough, pleural effusion, and evidence of malabsorption. For our patient, Whipple's disease is a unifying diagnosis for many seemingly unrelated symptoms across multiple organ systems. Whipple's disease can be fatal without appropriate treatment, but once diagnosed, it can be successfully treated with antibiotics [2]. It can be a difficult diagnosis to make, given its broad range of non-specific symptoms as well as the overall rarity of the disease. Physicians need to maintain a high level of clinical suspicion for the disease so that patients can be diagnosed and treated promptly.

## Case presentation

**Figure 1:**
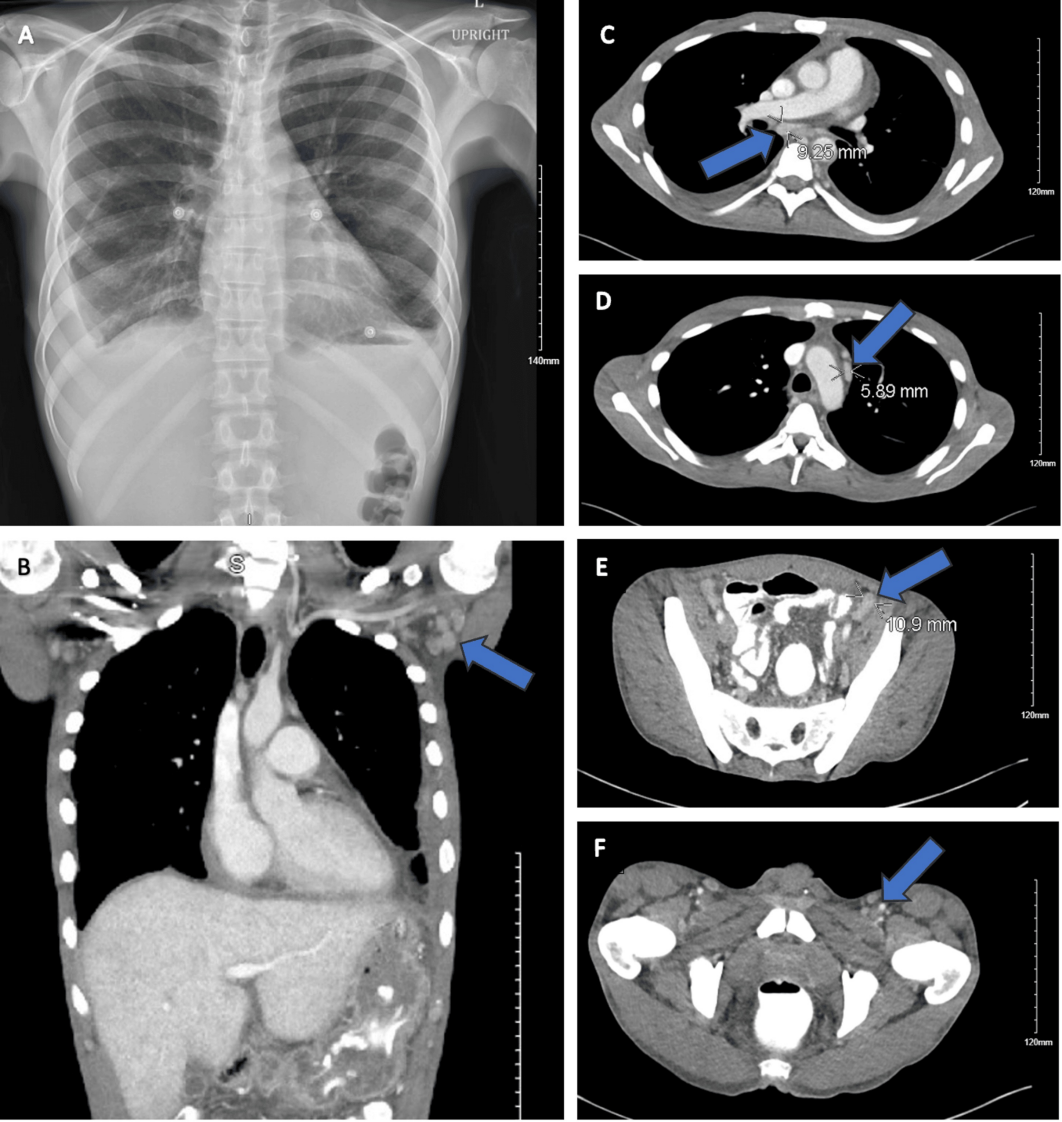
Imaging Findings (A) Bilateral pleural effusion; (B) Axillary lymphadenopathy; (C) Subcarinal-right hilar lymph node measuring 9.25 mm; (D) Prevascular lymph node measuring 5.89 mm; (E) External iliac chain lymph node measuring 10.9 mm; (F) Inguinal lymphadenopathy

We present the case of a 39-year-old male diagnosed with Whipple's disease with multiple organ system involvement. His past medical history includes chronic hepatitis B infection for which he was receiving entecavir. The patient presented to the hospital with a chief complaint of progressive generalized weakness and fatigue, early satiety, and a 50-pound unintentional weight loss over a two-month period. He also endorsed intermittent arthralgias and chronic cough. An outpatient CT scan of the chest, abdomen, and pelvis showed bilateral pleural effusion (also seen on chest X-ray) as well as axillary, mediastinal/hilar, and inguinal lymphadenopathy (Figure 1). Labs done by his primary care physician (PCP) in the months leading up to admission revealed elevated immunoglobulin G (IgG) and immunoglobulin A (IgA), low immunoglobulin M (IgM), elevated cytoplasmic anti-neutrophil cytoplasmic antibodies (c-ANCA), antinuclear antibody (ANA) speckled pattern, negative serum protein electrophoresis (SPEP), normal complement component 3 (C3) and complement component 4 (C4) levels and negative HIV testing. Negative fecal calprotectin made IBD less likely. Negative anti-endomysial antibodies and negative anti-tissue transglutaminase antibodies made celiac disease unlikely.

**Figure 2:**
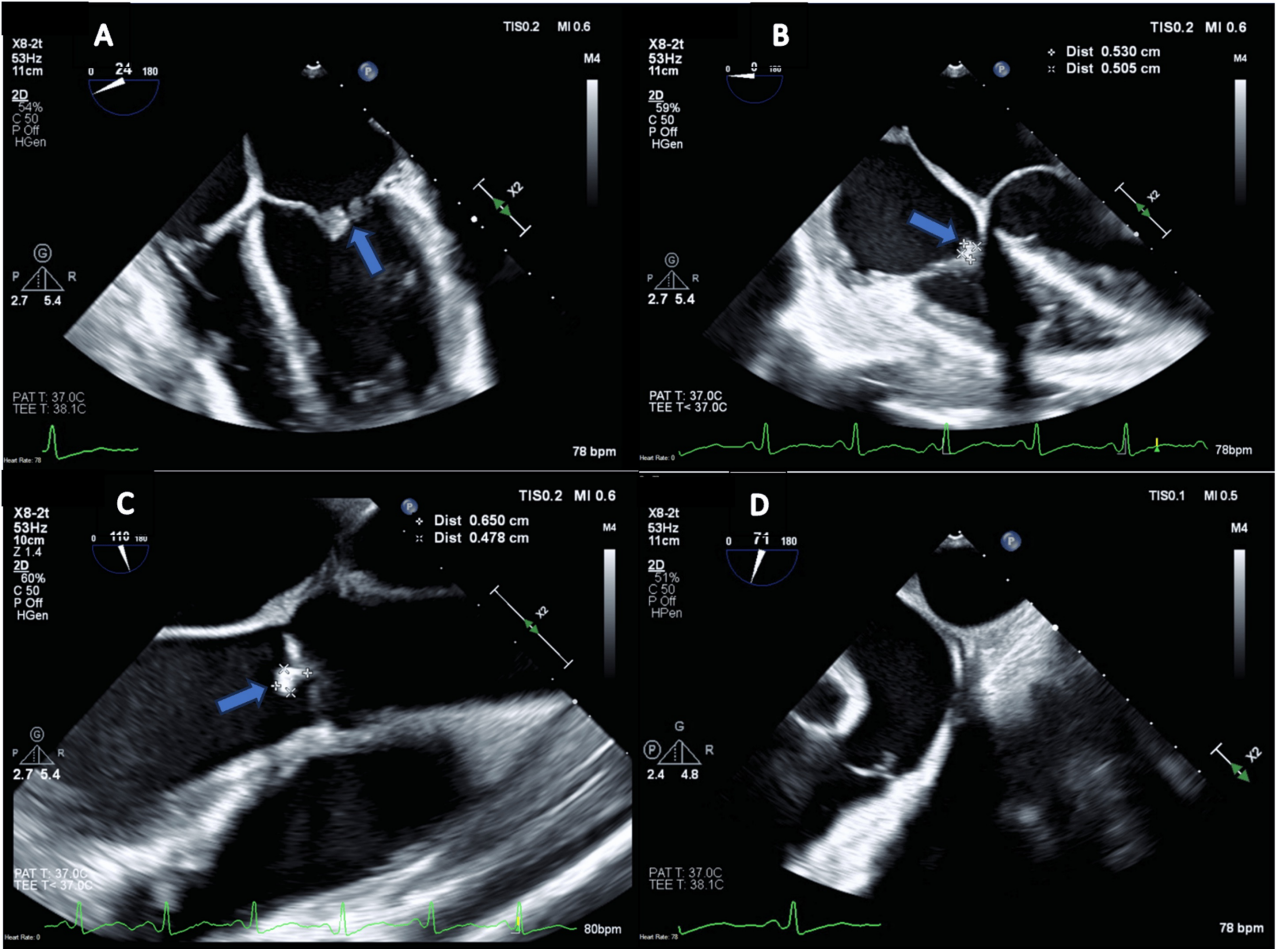
Echocardiographic Findings (A) Mitral valve vegetation; (B) Tricuspid valve vegetation; (C) Aortic valve vegetation; (D) Normal pulmonic valve

Vitals at presentation were notable for a temperature of 97.7°F, heart rate of 85 beats per minute, respiratory rate of 16 breaths per minute, and oxygen saturation (SpO_2_) of 100% on room air. Labs were significant for a sodium level of 127 mmol/L with otherwise unremarkable basic metabolic panel (BMP), normal liver function tests (LFTs), albumin 2.6 2.6 g/dL, WBC of 7 × 10^9^/L, hemoglobin (Hb) of 8.2 g/dL with microcytosis, and a platelet count of 92 × 10^9^/L. C-reactive protein (CRP) was elevated at 1.7 mg/dL, and the erythrocyte sedimentation rate (ESR) was at 100 mm/hr. Vitamin D, zinc, and iron levels were all low, with normal vitamin B12 and folate. Hepatitis B viral load was undetectable. Ova and parasite studies were negative as was testing for *Campylobacter*, *Salmonella, *and Shiga toxin *Escherichia coli*. The *Helicobacter pylori* stool antigen was positive, and the patient was started on appropriate treatment. TB testing was negative. Flow cytometry of peripheral blood smear revealed a small clonal CD5 positive B cell lymphoproliferative disorder. He had an echocardiogram done as part of a syncopal workup, and this revealed vegetations on the tricuspid (multiple vegetations, the largest measuring 11 × 4 mm), mitral (11 × 9 mm), and aortic (10 × 5 mm) valves (Table 1 and Figure 2). Blood cultures were negative. Workup for culture-negative endocarditis revealed a serum PCR positive for *T. whipplei.*

**Table 1:** Laboratory Values Ig: immunoglobulin; c-ANCA: cytoplasmic antineutrophil cytoplasmic antibody; ANA: antinuclear antibody; HIV: human immunodeficiency virus; BUN: blood urea nitrogen; WBC: white blood cell count; C3: complement component 3; C4: complement component 4

Laboratory Test (Units)	Patient Value	Reference Range
IgG (mg/dL)	3211	650-1600
IgA (mg/dL)	1245	40-350
IgM (mg/dL)	40.4	50-300
c-ANCA	1:80	-
ANA	1:160, speckled pattern	-
C3 (mg/dL)	90	90-200
C4 (mg/dL)	36	12-40
HIV 1 and 2	Non-reactive	Non-reactive
Fecal calprotectin (mcg/g)	11.7	<49.9
Endomysial antibody	<1:10	-
Tissue transglutaminase IgA antibody (IU/mL)	2.8	<6.9
Sodium (mEq/L)	127	135-145
Potassium (mEq/L)	4.2	3.4-5.0
Chloride (mEq/L)	97	95-110
Bicarbonate (mEq/L)	22	20-31
BUN (mg/dL)	7	6-25
Creatinine (mg/dL)	0.76	0.50-1.20
Aspartate aminotransferase (IU/L)	29	5-45
Alanine aminotransferase (IU/L)	<7	5-55
Alkaline phosphatase (IU/L)	69	30-120
Total bilirubin (mg/dL)	0.3	0.2-1.5
Albumin (g/dL)	2.6	3.5-5.2
WBC (K/mm³)	7	4.5-11
Hemoglobin (g/dL)	8.2	13.5-17.5
Mean corpuscular volume (fL)	74	80-100
Platelets (K/mm³)	92	150-450
C-reactive protein (mg/dL)	1.7	0-0.5
Erythrocyte sedimentation rate (mm/Hr)	100	0-15
Vitamin D (ng/mL)	22.89	30-100
Zinc (mcg/dL)	36	80-120
Ferritin (ng/mL)	266	10-307
Iron level (mcg/dL)	10	35-160
Total iron binding capacity (mcg/dL)	201	220-440
Percent saturation (%)	5	20-50
Vitamin B12 (pg/mL)	1030	211-911
Folate (ng/mL)	8.61	>5.39
Hepatitis B viral load	Not detected	Not detected
QuantiFERON-TB	Negative	Negative

**Figure 3:**
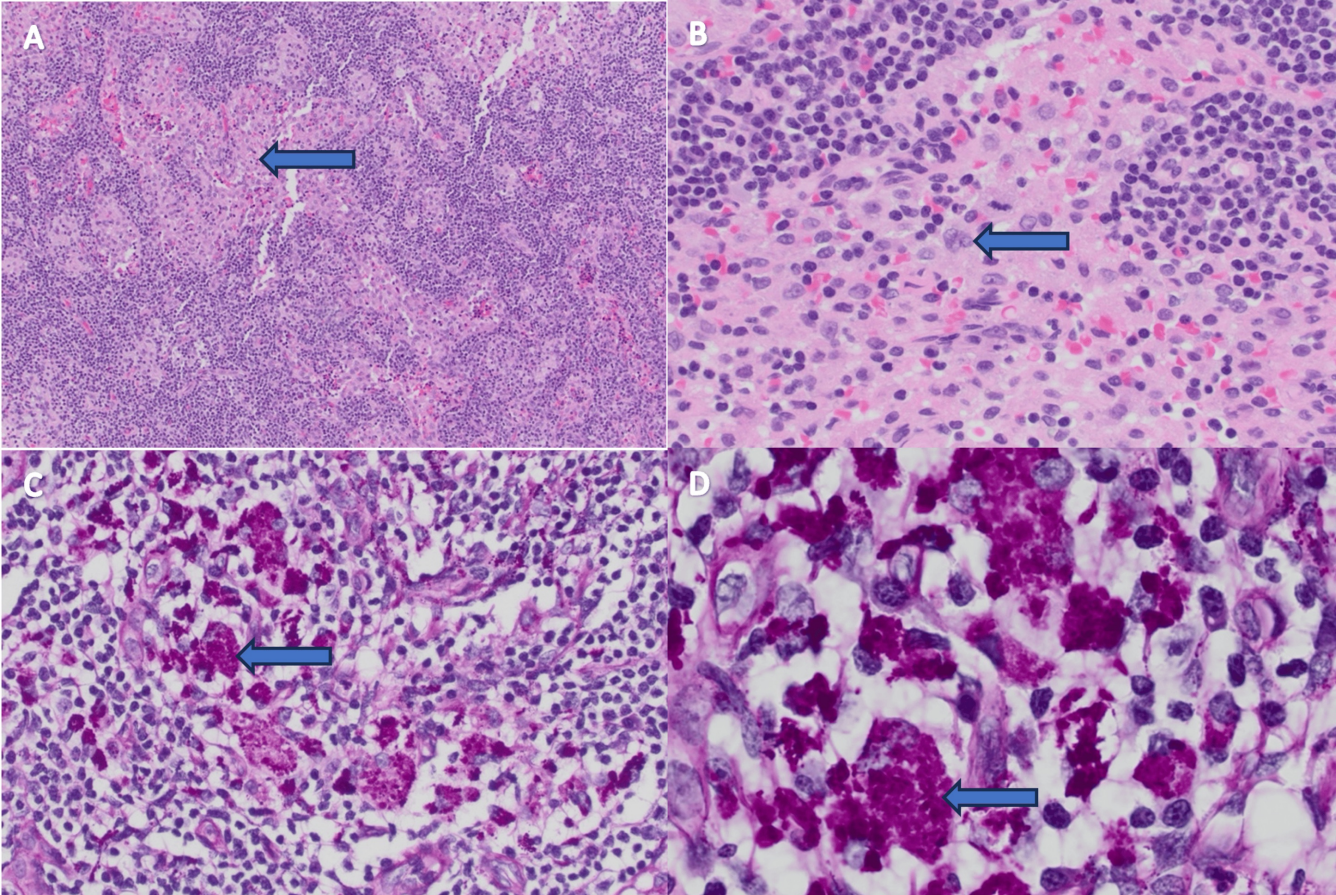
Inguinal Lymph Node Biopsy Findings (A) Sinus hyperplasia (hematoxylin and eosin (H&E) stain); (B) Foamy macrophages (H&E stain); (C) Periodic acid-Schiff with diastase (PAS/D) stain showing *Tropheryma whipplei* at 40× magnification; (D) PAS/D stain showing *T. whipplei* at 100× magnification *T. whipplei* has a plasma membrane enclosed by a three-layered cell wall. The inner layer of the cell wall contains polysaccharides that stain positive with PAS. When the organism is ingested by macrophages, the rod-shaped bacteria become visible within macrophages under PAS staining.

Due to the lymphadenopathy seen on his CT scan and the findings on flow cytometry, he underwent an inguinal lymph node biopsy, which revealed sinus hyperplasia with PAS-positive material in macrophages, highly suspicious for Whipple’s disease (Figure 3). There was no morphologic or immunophenotypic evidence of a lymphoproliferative disorder.

**Figure 4:**
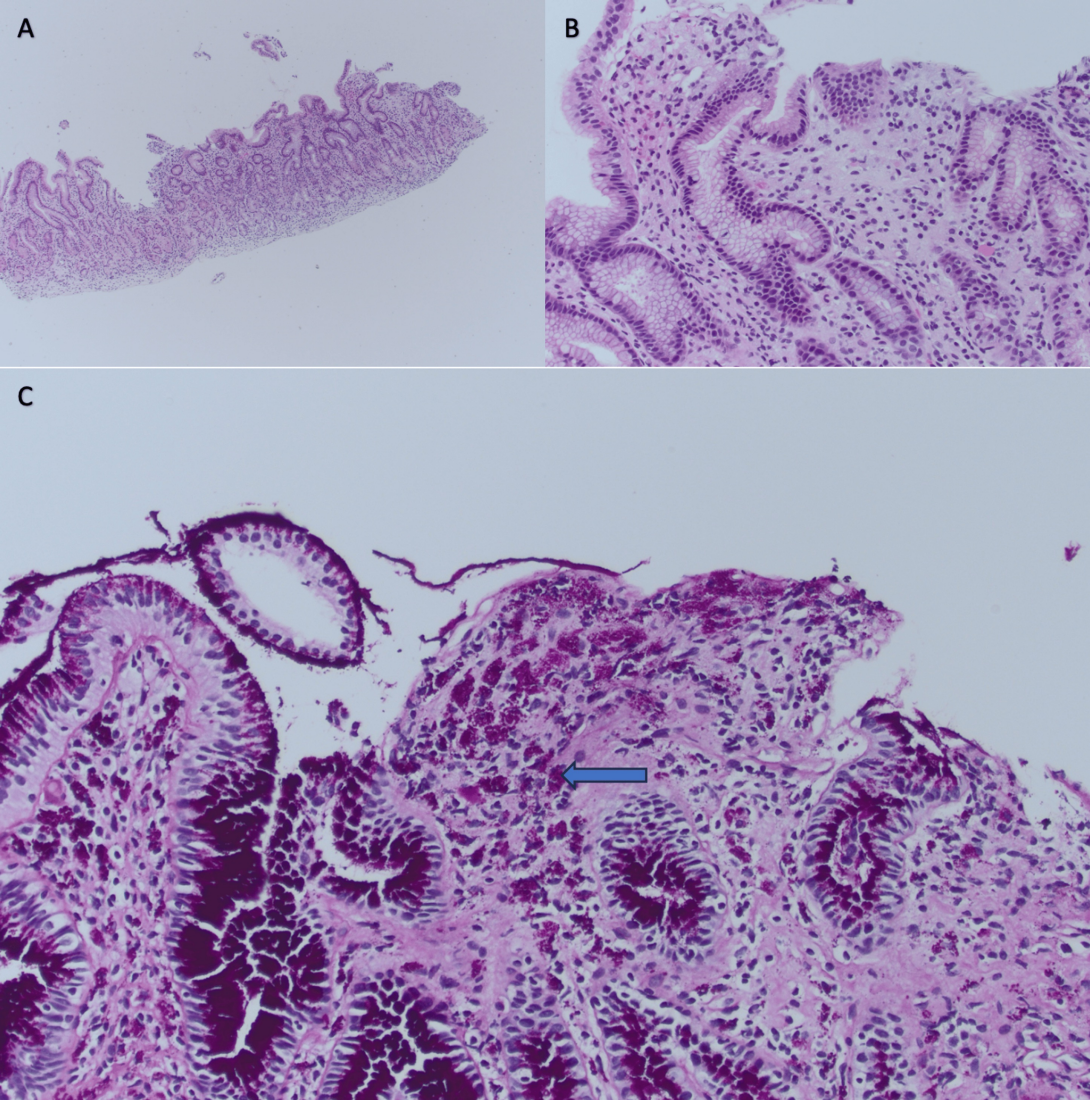
Gastric Antral Biopsy Findings (A) Hematoxylin and eosin (H&E) stain, low power; (B) H&E stain, high power; (C) Periodic acid-Schiff with diastase (PAS-D) stain, high power, showing PAS-positive macrophages in the antral mucosa *Tropheryma whipplei* has a plasma membrane enclosed by a three-layered cell wall. The inner layer of the cell wall contains polysaccharides that stain positive with PAS. When the organism is ingested by macrophages, the rod-shaped bacteria become visible within macrophages under PAS staining.

He had an esophagogastroduodenoscopy (EGD) and colonoscopy to rule out gastrointestinal (GI) malignancy. A colonoscopy showed a 1 cm polyp but was otherwise unremarkable. EGD revealed focal gastritis with pathology showing scattered PAS-positive macrophages in the antral mucosa, suspicious for Whipple’s disease (Figure 4). No *Helicobacter* organisms were identified. The patient was started on ceftriaxone 2 g IV daily for a four-week course and then will be transitioned to Bactrim DS for one year to complete treatment for his *T. whipplei* infection.

## Discussion

Infection with *T. whipplei *classically presents with the four cardinal symptoms of arthralgias, weight loss, diarrhea, and abdominal pain. Our patient had two of these symptoms, including arthralgias and weight loss, but lacked the abdominal pain or diarrhea that is classic in Whipple’s disease. In retrospect, the patient also had multiple other manifestations spanning multiple organ systems, including the seemingly unrelated symptoms of endocarditis, lymphadenopathy, cough/pleural effusions, and malabsorption syndrome. Whipple’s disease serves as a unifying diagnosis that explains all of his multiple organ system symptoms.

The most common initial presenting symptom of Whipple’s disease is arthralgia, which is present in almost 90% of cases [2-4]. Interestingly, arthralgia has been documented for more than a year (sometimes up to six years) before diagnosis [2-4]. Our patient initially presented with arthralgias over a year prior to being diagnosed with Whipple’s disease. He underwent extensive autoimmune workup outpatient, and his joint symptoms were presumed to be from polyarteritis nodosa in the setting of his hepatitis B infection. Arthralgia does not automatically prompt most clinicians to think about Whipple’s disease, and often, the early symptoms of arthralgia that are associated with Whipple’s disease are diagnosed as seronegative rheumatoid arthritis or other rheumatologic diseases. Given that arthralgia is such a common early manifestation of this rare disease, it is important that this presenting symptom prompt consideration of Whipple’s disease, especially in patients who do not respond to treatment for a presumed rheumatologic disorder.

*T. whipplei *infection is an important cause of culture-negative endocarditis and is being recognized with increasing frequency with improved molecular techniques [4]. A literature review from 2018 revealed 169 patients with Whipple’s endocarditis [4]. Twenty-three percent of these patients had involvement of multiple valves, with the aortic valve being the most commonly involved, followed by mitral and then tricuspid [4]. There were no documented cases of pulmonic valve involvement [4]. Our patient represents a case of triple valve involvement with vegetation on the aortic, tricuspid, and mitral valves. Our patient’s pulmonic valve was spared, consistent with a literature review of other cases of Whipple’s disease. Most cases of endocarditis from *T. whipplei* infection present with isolated endocarditis without any other clinical manifestations of Whipple’s disease [2]. Our patient is unique in that he presented with both endocarditis as well as multiple other manifestations of his disease.

About 50-60% of patients with Whipple’s disease present with lymphadenopathy, which raises concern for lymphoma [1-2,5-8]. Mesenteric and hilar/mediastinal lymphadenopathy are most common [1-2,5-8]. Retroperitoneal and peripheral lymphadenopathy have also been reported, sometimes as the sole manifestation [2,8,9]. It is not uncommon for Whipple’s disease to masquerade as malignancy, which was the case for our patient. Our patient presented with peripheral (axillary, inguinal) lymphadenopathy and mediastinal/hilar lymphadenopathy. The flow cytometry report was concerning for lymphoproliferative disorder, so lymph node biopsy was pursued. The biopsy showed PAS-positive material in macrophages, consistent with Whipple’s disease rather than lymphoproliferative disorder. In his case, lymphadenopathy was not the sole manifestation of his disease but rather a part of a constellation of symptoms.

Whipple’s disease also has the potential for pulmonary involvement and is known to cause chronic cough [2,10]. Imaging can reveal anything from pleural effusion to pulmonary nodules to interstitial changes or patchy infiltrates [10]. Pulmonary nodules appear to be the most common manifestation of pulmonary involvement [10]. Our patient presented with a chronic cough as well as bilateral pleural effusions on imaging. Both his cough and pleural effusion were suspected to be one of his many manifestations of Whipple’s disease.

Patients with Whipple’s disease present with weight loss and low serum albumin and are often found to have iron deficiency anemia and other vitamin/mineral deficiencies as a result of malabsorption [1,8,11]. Although Whipple’s disease is rare, it should be considered in the differential diagnosis of patients presenting with malabsorption syndrome after more common causes have been ruled out. In our patient, who presented with significant weight loss associated with low serum albumin, microcytic anemia with iron deficiency, as well as vitamin D deficiency and zinc deficiency, testing for more common causes of malabsorption syndrome was negative, leaving Whipple’s disease as the likely etiology.

## Conclusions

This clinical case demonstrates Whipple’s disease presenting as a wide array of symptoms across multiple organ systems, although, unfortunately, it lacks long-term follow-up on the patient's response to treatment. Our patient’s symptoms spanned from the classic GI manifestation of weight loss and malabsorption to musculoskeletal symptoms to pulmonary and cardiac involvement. The patient’s initial arthralgias were suspected to be from polyarteritis nodosa in the setting of his hepatitis B infection. His weight loss and poor appetite, along with lymphadenopathy, initially prompted concern for malignancy. The discovery of his culture-negative endocarditis finally raised suspicion for Whipple’s disease. Once this diagnosis was confirmed, it served to connect the dots between his multiple organ system symptoms. Although Whipple’s disease is rare, it should remain in the differential in patients with fitting symptoms so that it can be considered earlier in the disease process, allowing patients to receive more timely diagnosis and treatment. Once more common differential diagnoses have been ruled out with appropriate laboratory testing, PCR testing for Whipple's disease and, if indicated, endoscopic biopsy with PAS staining should be considered to help make the diagnosis.
